# Pathology caused by persistent murine norovirus infection

**DOI:** 10.1099/vir.0.059188-0

**Published:** 2013-11-13

**Authors:** Amita Shortland, James Chettle, Joy Archer, Kathryn Wood, Dalan Bailey, Ian Goodfellow, Barbara A. Blacklaws, Jonathan L. Heeney

**Affiliations:** 1 Department of Veterinary Medicine, University of Cambridge, Madingley Road, Cambridge CB3 OES, UK; 2 Nuffield Department of Surgical Sciences, University of Oxford, Level 6, John Radcliffe Hospital, Headington, Oxford OX3 9DU, UK; 3 Institute of Biomedical Research, University of Birmingham, Birmingham B15 2TT, UK; 4 Division of Virology, Department of Pathology, University of Cambridge, Addenbrooke’s Hospital Level 5, Hills Road, Cambridge CB2 2QQ, UK

## Abstract

Subclinical infection of murine norovirus (MNV) was detected in a mixed breeding group of WT and *Stat1*^−/−^ mice with no outward evidence of morbidity or mortality. Investigations revealed the presence of an attenuated MNV variant that did not cause cytopathic effects in RAW264.7 cells or death in *Stat1*^−/−^ mice. Histopathological analysis of tissues from WT, heterozygous and *Stat1*^−/−^ mice revealed a surprising spectrum of lesions. An infectious molecular clone was derived directly from faeces (MNV-O7) and the sequence analysis confirmed it was a member of norovirus genogroup V. Experimental infection with MNV-O7 induced a subclinical infection with no weight loss in *Stat1*^−/−^ or WT mice, and recapitulated the clinical and pathological picture of the naturally infected colony. Unexpectedly, by day 54 post-infection, 50 % of *Stat1*^−/−^ mice had cleared MNV-O7. In contrast, all WT mice remained infected persistently. Most significantly, this was associated with liver lesions in all the subclinically infected WT mice. These data confirmed that long-term persistence in WT mice is established with specific variants of MNV and that despite a subclinical presentation, active foci of acute inflammation persist within the liver. The data also showed that STAT1-dependent responses are not required to protect mice from lethal infection with all strains of MNV.

## INTRODUCTION

Understanding the mechanisms of viral persistence of non-integrating RNA viruses will facilitate ultimately their control. Persistence of members of the family *Caliciviridae* has been reported after infection with feline calicivirus (FCV), rabbit haemorrhagic disease virus (RHDV), and human and murine norovirus (HuNoV and MNV) (Capizzi *et al.*, 2011; Coyne *et al.*, 2006; Forrester *et al.*, 2003; Hsu *et al.*, 2006; Siebenga *et al.*, 2008; Thackray *et al.*, 2007). Studies on HuNoV persistence have focused on mechanisms of viral evolution, antigenic variation and receptor switching to allow persistence in the population, but if and how ‘within-host’ persistence occurs is not understood clearly. Persistence of FCV and RHDV is thought to occur by gradual mutation driven by the immune response, but recombination between strains and reinfection may also occur (Coyne *et al.*, 2007; Forrester *et al.*, 2008).

MNV has been reported to persist in both immunocompromised and immunocompetent mice, and persistent infection can occur in immunocompetent hosts despite robust seroconversion (Hsu *et al.*, 2006; Karst *et al.*, 2003; Thackray *et al.*, 2007). Acute and persistent strains of MNV belong to the same genogroup (V), and serogroup and systemic infection occurs with both types, with spread to the spleen, liver and lungs as well as other organs (Hsu *et al.*, 2006; Thackray *et al.*, 2007). A role for antibodies and T-cells in the clearance of acute MNV from tissues and the intestine has been demonstrated (Chachu *et al.*, 2008a, b), but persistent MNV strains are less well characterized, and there are few comparisons between acute and persistent strains (Kahan *et al.*, 2011; Nice *et al.*, 2013).

How persistent norovirus infection is maintained is unclear. Changes in surface antigens may enable evolving viral progeny to evade the immune system, facilitating their persistence in the host. This has been shown for FCV and MNV during chronic infection where mutations occur in areas predicted to be important for immune recognition (Arias *et al.*, 2012; Johnson, 1992; Radford *et al.*, 1998). Impairment of immune cell function may be another way in which virus can persist. MNV replicates in macrophages and dendritic cells and, like many viruses that infect antigen-presenting cells, may also impair the activation of T-cells (Tomov *et al.*, 2013) and B-cells (Oldstone, 2006; Wobus *et al.*, 2004). Studies with two persistent strains of MNV have implicated colonic tropism in persistent infection of immunocompetent mice; however, the immunopathological consequences of infection by these viruses have not been established (Arias *et al.*, 2012; Nice *et al.*, 2013).

Here, we report on the identification and characterization of a variant of MNV (MNV-O7) derived from a small group of mixed breeding animals consisting of *Stat1*^−/−^ and WT mice that had no clinical abnormalities or increased mortality. Surprisingly, all animals in this group were infected subclinically. Virus growth was noncytopathic *in vitro*. Furthermore, although experimental infection of immunocompetent and *Stat1*^−/−^ mice was subclinical, histopathology revealed a progressive, subclinical multifocal hepatitis. An infectious molecular clone was derived directly from faeces, which recapitulated this pathology. Interestingly, infection of immunocompetent mice showed that all animals had long-term persistence of MNV-O7, whilst resolution and clearance occurred in 50 % of *Stat1*^−/−^ mice. Here, we report on the characterization of a non-pathogenic MNV strain in *Stat1* knockout mice with direct derivation of an infectious molecular clone from faeces.

## RESULTS AND DISCUSSION

### Natural MNV-O7 infection is subclinical in *Stat1*^−/−^ mice

MNV was first described as an acute infection with mortality and morbidity apparent in *Stat1*^−/−^ mice, persistent subclinical infection in *Rag*^−/−^ mice, and acute subclinical infection in WT mice (Karst *et al.*, 2003). STAT1 (signal transducer and activator of transcription-1) is a central molecule in signalling from all IFN receptors and as such is key in inducing the innate immune response antiviral pathways. RAGs are the recombination activating enzymes involved in B- and T-cell receptor rearrangement, and mice without the genes for these proteins do not have an adaptive immune response. It was therefore proposed by Karst *et al.* (2003) that the IFN pathway is important in controlling the level of MNV infection, but that adaptive immune responses are important in clearing the virus. MNV has also been reported as causing a persistent infection in immunocompetent mice (Godinez *et al.*, 2009; Hsu *et al.*, 2006, 2007; Karst *et al.*, 2003; Manuel *et al.*, 2008; Thackray *et al.*, 2007). To date, however, there has been no report of the consequences of subclinical and persistent MNV infection in *Stat1*^−/−^ mice.

A mixed colony of *Stat1*^−/−^ mice with WT and heterozygous mice was found to be seropositive for MNV by diagnostic ELISA. However, all mice were healthy and the high morbidity or mortality seen previously in *Stat1*^−/−^ mice (Karst *et al.*, 2003; Wobus *et al.*, 2004) was not evident. The genotype of the mice was confirmed using two different sets of published primers (Agrawal *et al.*, 2007; Mohan *et al.*, 2000) (data not shown). Surprisingly, at necropsy all the *Stat1*^−/−^, 63 % of the heterozygous and none of the WT mice had splenomegaly. In addition, *Stat1*^−/−^ mice had multifocal, round slightly raised pale foci throughout the liver parenchyma (data not shown). Occasionally, the mesenteric lymph nodes were also enlarged, but no other gross abnormalities were detected.

**Fig. 1:**
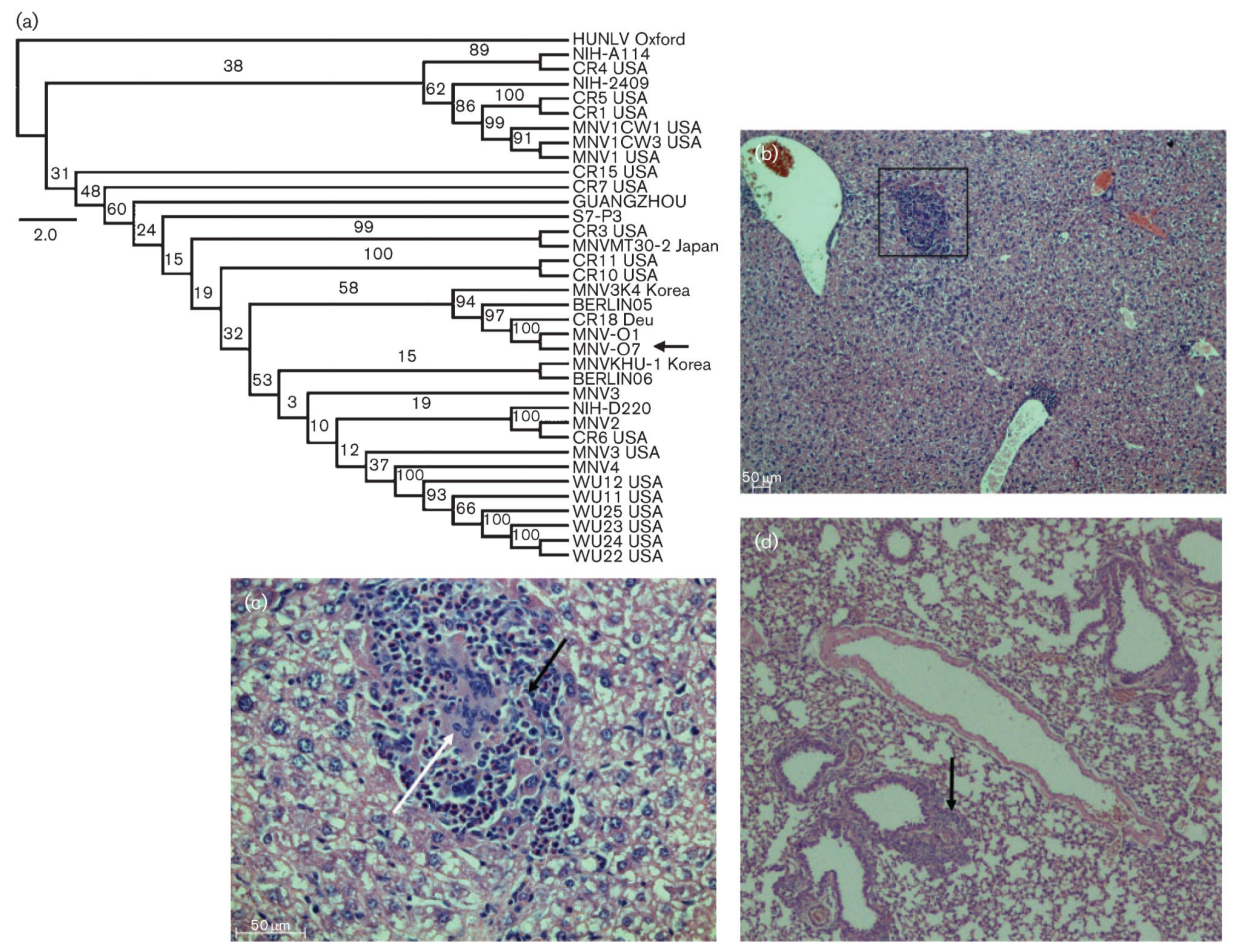
MNV phylogenetic tree including MNV-O7 and pathology caused by natural infection. (a) Full-length genomic MNV nucleotide sequences were used to construct a maximum-likelihood phylogenetic tree with PhyML and 100 bootstrap interactions. The bootstrap values are indicated on the branches. Bar, 2 % nucleotide divergence. (b–d) Representative pathology in liver (b, c) and lung (d) sections from naturally infected *Stat1*^−/−^ mice from which MNV-O7 was derived. (b) A focus of inflammation in the liver parenchyma that is magnified in (c) to show the presence of inflammatory cells (macrophages shown by the white arrow) and necrotic (black arrow) cells. (d) General infiltration of the lung; the arrow indicates a localized focus of inflammation associated with a bronchiole and blood vessel. Original magnification, ×10 (b, d) and ×40 (c).

Histopathology revealed lesions in the liver (*Stat1*^−/−^, Fig. 1b, c; heterozygous and WT mice, Fig. S1, available in JGV Online), spleen, intestine and lungs (data not shown); these varied from normal to mild/moderate foci of inflammation and in 33 % of the mice multifocal to diffuse necrosis. A vasculitis (Fig. S1a) with occasional mild inflammatory foci occurred in the liver parenchyma of 15 % of the WT and 56 % of the heterozygous mice (data not shown), whilst the livers of 22 % of the heterozygous and 83 % of the *Stat1*^−/−^ mice showed more severe multifocal to diffuse areas of inflammation often accompanied by necrosis and fibrosis (Figs 1c and S1b). Lesions were present in the spleens, and varied from red pulp hyperplasia and activation of the white pulp in both WT and heterozygous mice (20 and 38 %, respectively) to multifocal inflammation and necrosis in 50 % of the heterozygous and all of the *Stat1*^−/−^ mice (data not shown). In the lungs, 73 % of the *Stat1*^−/−^ mice had evidence of pneumonia with focal to multifocal perivascular inflammatory cell infiltrates (Fig. 1d).

In WT mice naturally infected with this strain of MNV (referred to as MNV-O7), enlargement of Peyer’s patches with increased germinal centres was observed. In *Stat1*^−/−^ mice, mesenteric lymph nodes were enlarged with expanded, hyperplastic germinal centres (data not shown).

### Direct isolation of a full-length molecular clone of MNV-O7 - a unique MNV strain

In order to characterize the virus without adaptation in tissue culture, an infectious full-length molecular clone called MNV-O7mc was derived directly from faeces of a *Stat1*^−/−^ mouse. Western blotting of the supernatant of molecular clone-transfected cells revealed expression of MNV-O7mc RNA polymerase (Fig. S2). Reverse transcriptase (RT)-PCR using RNA extracted from RAW264.7 cells infected with supernatant from the transfection produced a strong band at 186 bp as expected for MNV-1 and MNV-O7 (data not shown). This confirmed *in vitro* that the reverse genetics system had produced infectious virus.

MNV-O7mc was sequenced. A phylogenetic tree of the predicted amino acid sequences of VP1 showed that MNV-O7 clustered with other MNV strains in genogroup V separate from norovirus genogroups I, II, III and IV (data not shown), in agreement with previous reports (Thackray *et al.*, 2007). The predicted VP1 amino acid sequence of MNV-O7 was <11 % divergent compared with other MNV strains, which supported the classification of MNV-O7 within genogroup V. Phylogenetic analysis of MNV genomic nucleotide sequences showed MNV-O7 to cluster with MNV-O1 (isolated from a WT mouse in the same colony), on a branch separate from other MNV strains, but closely related to two German isolates (GV/CR18/2005/DEU and Berlin/05/O6/DE) and a South Korean isolate (MNV3/K4/2009/Korea) (Fig. 1a). This most probably reflects movement of mice between breeding and/or experimental animal facilities. MNV-O7 was identified as a unique strain as it diverged by >3 % from all other MNV strains (Thackray *et al.*, 2007).

MNV-O7 had four ORFs characteristic of MNV (Thackray *et al.*, 2007). Compared to MNV-1, MNV-O7 ORF2 had 178 nt differences (Fig. S3) corresponding to 17 aa differences, including a codon (CAA) deleted at nt 6676 as has been observed for CR18 (Thackray *et al.*, 2007). Phylogenetic analysis also showed CR18 to be related closely to MNV-O7 (Fig. 1a). The majority of the differences seen were in the P domain of the capsid (aa 229–537), which is consistent with the P domain being the most variable (Thackray *et al.*, 2007). However, the differences were spread across the P region and not restricted to the P2 domain (aa 278–415) of the capsid. MNV-O7 had a glutamic acid at aa 296 in the P2 region of VP1, which is known to be associated with avirulence in *Stat1*^−/−^ mice (Bailey *et al.*, 2008). The majority of MNV strains also have a predicted glutamic acid in this region of VP1, with only MNV-1 and its derivatives containing lysine at this position. The epitope for the neutralizing mAb A6.2 is also conserved in MNV-O7 (leucine at residue 386) (Katpally *et al.*, 2008; Lochridge & Hardy, 2007; Taube *et al.*, 2010).

Comparison of ORF1 between MNV-O7 and MNV-1 revealed 80 aa differences (Fig. S3). These included a glutamic acid at position 94 of NS1/2 that is associated with growth in the proximal intestine in the persistent strain CR6 (Nice *et al.*, 2013). ORF3 had 21 and ORF4 24 aa differences between MNV-O7 and MNV-1 (Fig. S3).

To prove that the MNV-O7mc was infectious *in vivo*, three *Stat1*^−/−^ and three WT mice received 1×10^4^ RNA copies of MNV-O7mc, whilst another three *Stat1*^−/−^ and three WT mice were mock inoculated. The mice were observed daily (for 7 days) and clinical signs were absent. A diagnostic RT-PCR for MNV carried out on faecal RNA collected pre-infection and at day 7 post-infection (p.i.) showed no product in the control animals before and after inoculation, and no product in the challenged mice before infection with MNV-O7mc (data not shown). Importantly, the faeces were virus-positive at day 7 p.i. for almost all animals inoculated with MNV-O7mc (three of three WT and two of three *Stat1*^−/−^ mice, data not shown). A MNV RT-quantitative (q)PCR assay on the samples showed that all of the challenged mice were infected, with viral titres in faeces ranging from 4×10^4^ to 4×10^5^ copies ml^−1^. The single *Stat1*^−/−^ mouse that was negative by diagnostic RT-PCR had a low viral copy number.

A separate group of WT mice was also infected with MNV-O7mc and the virus caused persistent infection (three of three mice shedding virus in faeces at day 30 p.i.).

The filtered supernatant of faecal homogenate from the original MNV-O7-positive *Stat1*^−/−^ mouse was incubated with RAW264.7 cells for 4 days, yet no cytopathic effects (CPEs) were visible (Fig. S4b), despite an increase in viral RNA measured by RT-qPCR. MNV-1 did cause CPEs (Fig. S4c). At day 4 p.i., viral RNA levels with MNV-O7 as detected by RT-qPCR were similar to MNV-1 levels. The MNV-O7mc stock grown in RAW264.7 cells was also noncytopathic (data not shown).

Our data illustrate that full-length amplification of infectious MNV without growth in cell culture is possible and can provide a method of characterizing non-cytopathic viruses. This method of producing infectious virus limits the mutations that might be caused by tissue culture passage and these are known to attenuate MNV-1 in *Stat1*^−/−^ mice (Bailey *et al.*, 2008; Wobus *et al.*, 2004). There has also been concern expressed that experimental infections showing persistence of MNV have only been carried out with virus grown in tissue culture that may select for this trait (Hsu *et al.*, 2007). These concerns have been addressed here to show that a directly isolated virus was attenuated in *Stat1*-knockout mice and caused persistent infections in immunocompetent mice. Production of a fulllength clone by RT-PCR may also induce RT- and PCR polymerase-related mutations; however, these can be minimized by the use of a proofreading PCR enzyme, as was used here.

### Control of MNV is not dependent on STAT1 responses

**Fig. 2:**
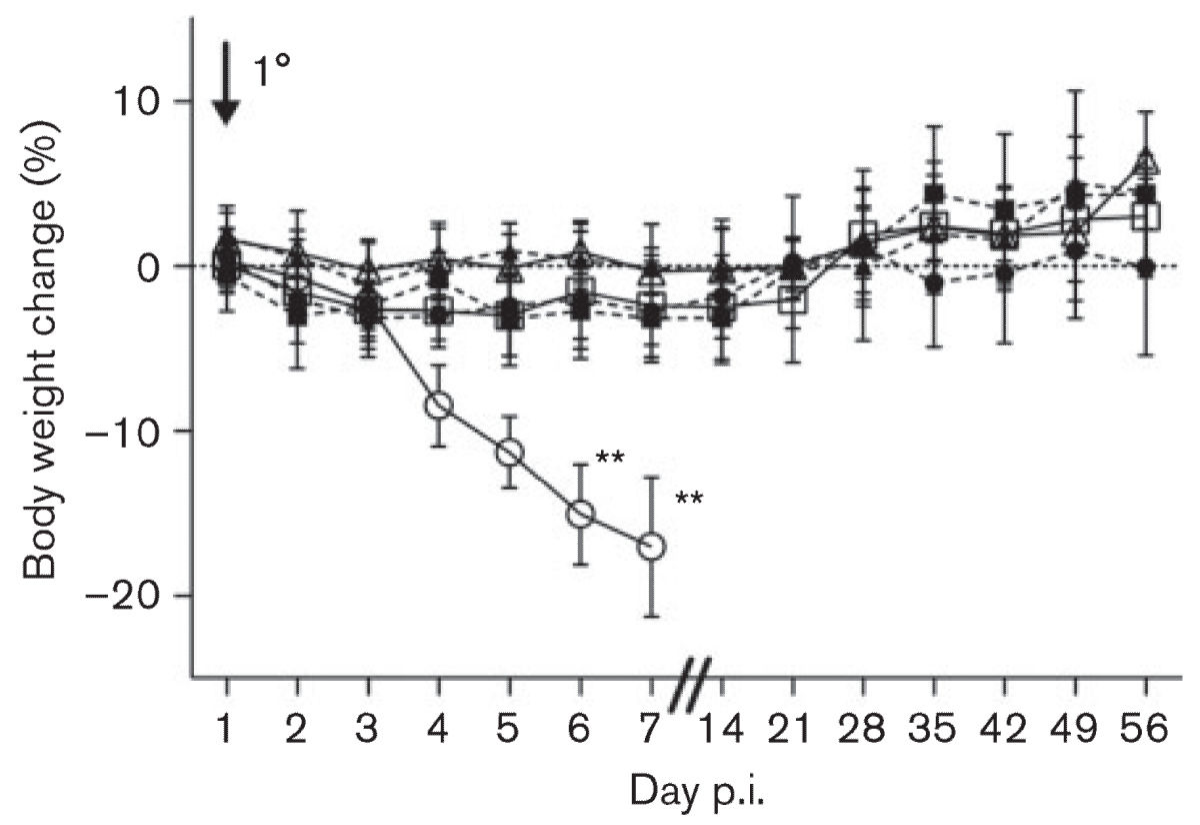
Percentage body weight change in mice infected with MNV. Mice (*n*=8) were mock-infected or infected with 10^8^ copies of MNV RNA by oral gavage on day 0, and the body weight change scored daily and then weekly (medians shown). Circles, MNV-1.CW3; squares, MNV-O7; triangles, mock-infected mice; open symbols, WT mice; closed symbols, *Stat1*^−/−^ mice. *P*-values were determined using one-way ANOVA and Dunnet’s post tests to compare between groups in the same genetic background; **P<0.01. Bars, sd.

MNV-1 was first identified because it caused mortality in *Rag2*^−/−^*Stat1*^−/−^ mice and this was shown to be due to the lack of STAT1. This finding implied an important role for STAT1-mediated immune responses in the control of MNV infection in mice (Karst *et al.*, 2003). Here, *Stat1*^−/−^ and WT mice were infected with 10^8^ RNA copies of MNV-1 and MNV-O7. All virus-infected animals were positive for MNV RNA in the faeces at day 5 p.i. (Table 1). The animals were observed daily for any clinical signs. There was a decrease in body weight in *Stat1*^−/−^ mice infected with MNV-1 compared with mock-infected *Stat1*^−/−^ mice at days 6 and 7 p.i. (*P*<0.01, Fig. 2). This confirmed that MNV-1 infection was virulent in *Stat1*^−/−^ mice. There was no difference in the weight gain between MNV-O7-infected *Stat1*^−/−^ mice, MNV-O7- and MNV-1-infected WT mice, and mock-inoculated mice on the same genetic background. At day 5 p.i., *Stat1*^−/−^ mice infected with MNV-1 showed a hunched posture, piloerection, subdued behaviour, distended abdomens and reduced faecal output, and by day 7 p.i. these mice had been euthanized due to the clinical signs, as expected (Karst *et al.*, 2003). In contrast, no clinical signs were observed in mock- or MNV-O7-infected *Stat1*^−/−^ mice, nor in mock- or virus-infected WT mice. This supports the hypothesis that MNV pathogenicity in *Stat1*^−/−^ mice is dependent on the viral strain. It also agrees with previous reports where 129 mice infected acutely by the intracerebral, intranasal and peroral routes with MNV-1 showed no clinical signs (Karst *et al.*, 2003). Other studies have shown faecal inconsistency soon after MNV-1 infection, but this has not been replicated with other MNV strains such as MNV-3 (Kahan *et al.*, 2011; Mumphrey *et al.*, 2007).

A diagnostic RT-PCR was performed on faecal and tissue samples from the animals remaining on study at day 54 p.i. (Table 1). No viral RNA was detected in the faecal samples from any of the *Stat1*^−/−^ mice infected with MNV-O7, although viral RNA was isolated from the spleens and livers of ~50 % of these mice. However, viral RNA was found in the faeces, liver and spleen from all the WT mice infected with MNV-O7. These data indicated that MNV-O7 was only secreted actively from persistently infected WT mice, with *Stat1*^−/−^ mice clearing virus RNA from the gut more rapidly than from the spleen and liver. Subsequent studies in our laboratory confirmed the presence of MNV-O7 RNA in the caecum, colon and mesenteric lymph node of chronically infected WT mice (data not shown). Infections with other persistent strains of MNV (MNV-2, -3 and-4) also produced detectable RNA in the faeces, mesenteric lymph node, jejunum, caecum and colon at 8 weeks p.i. (Arias *et al.*, 2012; Hsu *et al.*, 2006). In C57BL/6 mice, viral RNA has been detected in faeces for up to 9 months p.i. with MNV strains CR1, CR3, CR6 and CR7, and in addition in the mesenteric lymph nodes and distal ileum for CR1 and CR3 (McFadden *et al.*, 2013; Thackray *et al.*, 2007). However, in these studies no significant live viral titres were found. A discrepancy in virus detection using either RT-PCR or live virus assays has been reported, and could be due to the limit of live viral detection, the viral genome copy number being much higher than the actual number of replication-competent infectious virus particles or neutralizing antibody interfering with virus isolation (Thackray *et al.*, 2007).

All the WT mice infected with MNV-1 had cleared virus from faeces and tissues by day 54 p.i. (Table 1), which is in agreement with previous studies (Hsu *et al.*, 2006; Karst *et al.*, 2003). The inability of MNV-1 to cause persistent infection has now been attributed to the presence of aspartic acid at position 94 of NS1/2. This amino acid is glutamic acid in other isolated MNV strains, including MNV-O7. The MNV-1 mutation may have arisen by *in vitro* tissue culture adaptation or adaptation *in vivo* by intracerebral passage (Thackray *et al.*, 2007).

Taken together, we have shown that control of MNV infection was not dependent on STAT1 responses, as mice without the *Stat1* gene survived MNV-O7 infection. Normal IFN-induced responses were only important in controlling levels of replication with MNV-1, as has been shown previously (Karst *et al.*, 2003). It was surprising to find that MNV-O7 was cleared from all faecal samples and the tissues of ~50 % *Stat1*^−/−^ mice during chronic infection. Although STAT1 is the major transcription factor used to signal from IFN receptors, it is not the only factor used (Gil *et al.*, 2001; Malmgaard *et al.*, 2002; Ramana *et al.*, 2002; Shresta *et al.*, 2005). It may also mean that important regulators of immune signalling, e.g. the SOCS (suppressor of cytokine signalling) proteins, are not induced and so aberrant cytokine responses may be induced in these mice. Differences in cytokine responses between *Stat1*^−/−^ and WT mice would be worth exploring to determine which gene or genes may be driving the clearance of MNV in this genetic background.

### Gross spleen and liver pathology in MNV-O7-infected mice

After acute infection, splenomegaly was evident in MNV-1-infected *Stat1*^−/−^ mice, characterized by decreased coloration and an increase in length (*P*<0.05) compared with mock-infected and MNV-O7-infected mice (Fig. S5). There were no differences in the spleen size between mock-infected and MNV-1- or -O7-infected WT mice and mock-infected and MNV-O7-infected *Stat1*^−/−^ mice. Similarly, there were no differences in size or gross pathology of the livers between mock-infected and virus-infected WT and *Stat1*^−/−^ mice (data not shown). However, at day 54 p.i. (i.e. chronic infection), the spleens from *Stat1*^−/−^ mice infected with MNV-O7 were elongated (*P*<0.05) compared with mock infected *Stat1*^−/−^ mice and WT mice infected with MNV-O7 (Fig. S6). There was no significant difference in the spleen length between the other animal groups. The spleens from MNV-O7-infected *Stat1*^−/−^ mice had pale foci. At this time point, there was no difference in the size of the intact livers; however, pale foci were seen in the livers from *Stat1*^−/−^ mice infected with MNV-O7 (data not shown).

Previous studies have shown that spleens from *Stat1*^−/−^ mice infected with MNV were enlarged grossly and that sometimes the livers had pale spots, but whether these changes were acute or chronic was unclear (Ward *et al.*, 2006). The lack of gross pathological lesions after MNV infection in immunocompetent mice is in agreement with other studies (Perdue *et al.*, 2007).

### Histopathological lesions in MNV-O7-infected mice

**Fig. 3:**
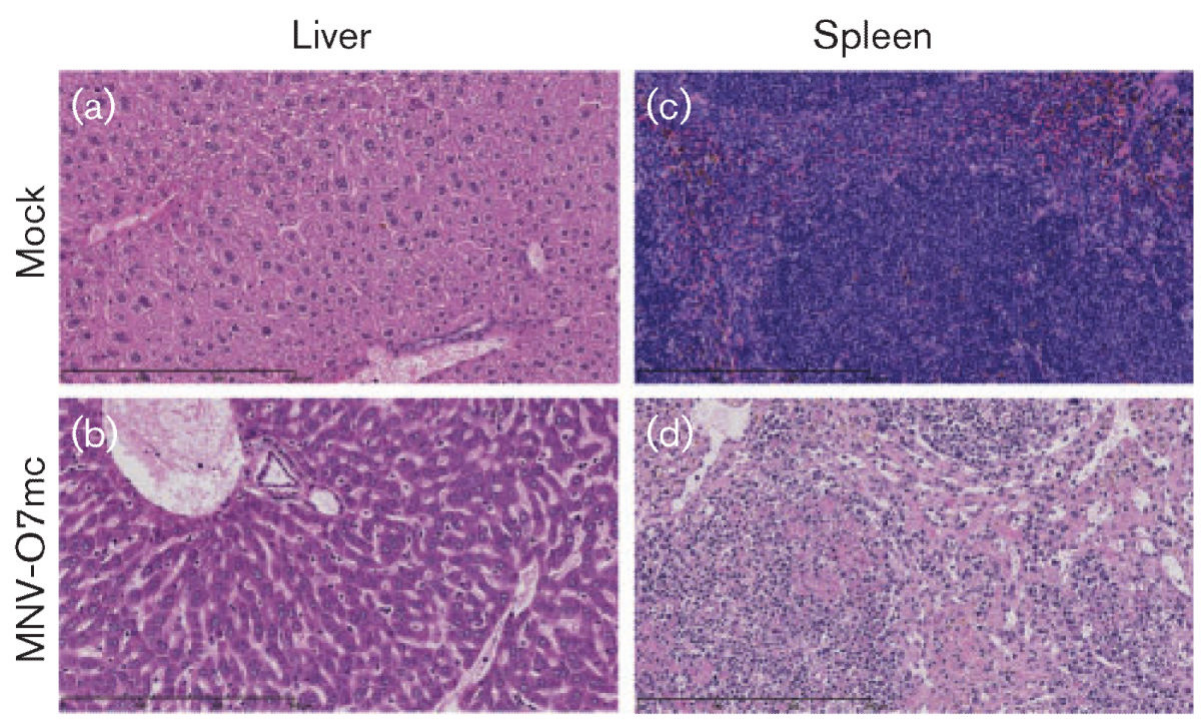
Liver and spleen pathology in *Stat1*^−/−^ mice infected with MNV-O7mc. Examples of liver and spleen pathology seen in mock- and MNV-O7mc (10^4^ RNA copies by oral gavage)-infected *Stat1*^−/−^ mice at day 7 p.i. (a, c) Normal tissue architecture in mock-inoculated animals. (b) A general increase in inflammatory cells and prominent cannuli. (d) An area of coagulative necrosis and pyknotic debris suggestive of apoptosis. Original magnification, ×20; bar, 300 μm.

**Fig. 4:**
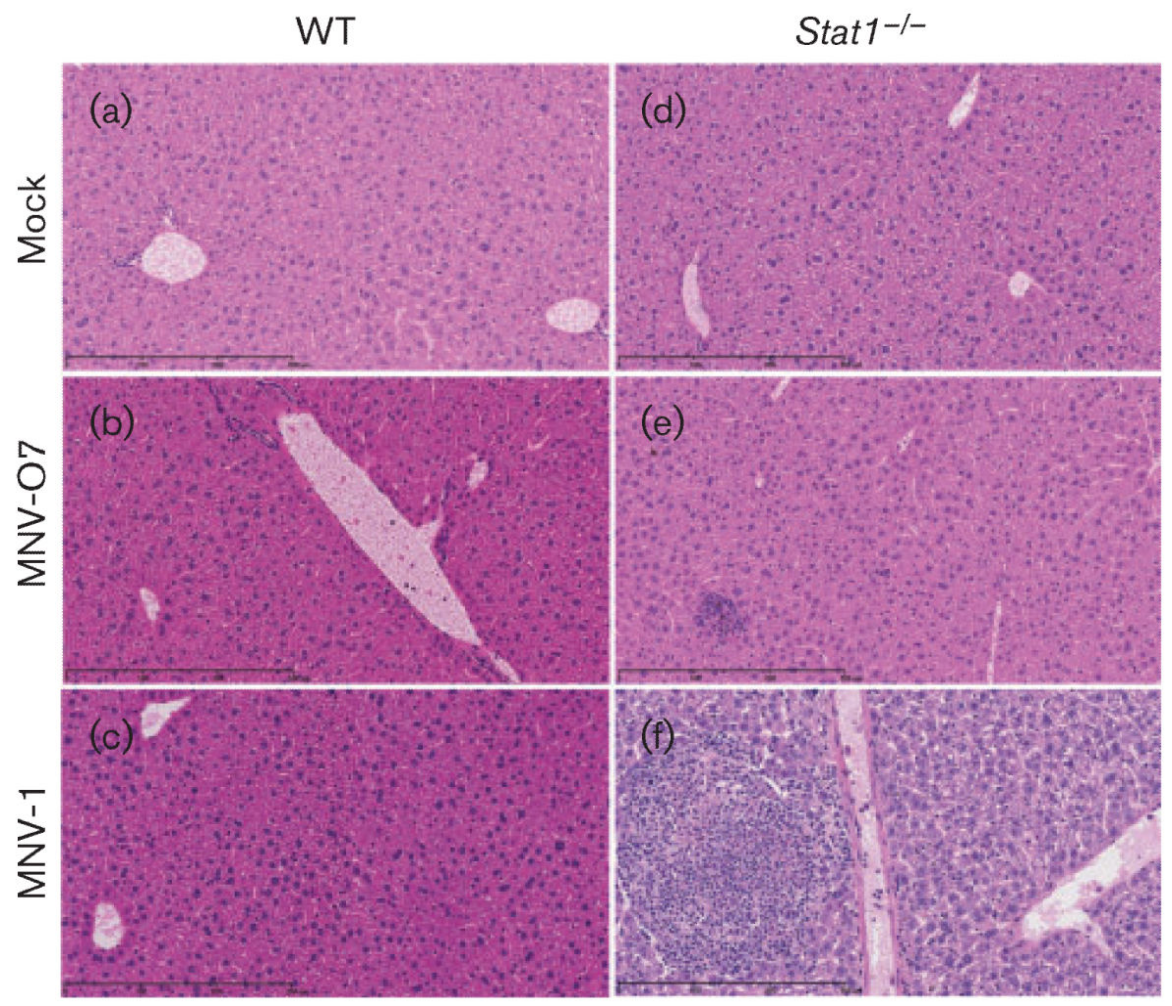
Histopathology in livers during acute MNV infection. Haematoxylin & eosin-stained liver sections from mock (a, d) or infected mice (10^8^ RNA copies by oral gavage) at day 5 p.i. [MNV-O7 (b, e) and MNV-1 (c, f)]; infected WT (a–c) and *Stat1*^−/−^ (d–f) mice are shown. Original magnification, ×20 bar, 300 μm.

Both the MNV-O7mc and tissue culture-derived virus caused histopathological lesions in the liver (Figs 3b and 4e) and spleen (Fig. 3d) of *Stat1*^−/−^ mice after acute infection, whilst mock-inoculated mice showed no liver or splenic pathology (Figs 3a and 4d, liver; Fig. 3c, spleen). The livers of *Stat1*^−/−^ mice infected with MNV-O7mc had prominent cannuli and an increase in inflammatory cells (Fig. 3b), and the spleens had areas of coagulative necrosis and pyknotic debris suggestive of apoptosis (Fig. 3d). At this acute time point, WT mice infected with MNV-O7mc and tissue culture-derived MNV-O7 had no histopathological lesions (Fig. 4b).

As in MNV-O7-infected immunocompetent and *Stat1*^−/−^ mice, acutely MNV-1-infected immunocompetent mice had mild-to-moderate reactive hyperplasia in the spleens (data not shown). At this time point the spleens from MNV-1-infected *Stat1*^−/−^ mice had diffuse reactive hyperplasia, expansion of the red pulp and activation of white pulp with the presence of pyknotic debris, suggesting apoptosis. In addition, two of these mice had focal areas of coagulative necrosis with a neutrophilic inflammatory infiltrate in the spleen (Fig. S7). There was moderate vasculitis and infiltration of inflammatory cells, mainly lymphocytes, in the liver from *Stat1*^−/−^ mice infected with MNV-1. Coagulative necrosis accompanied by inflammation was also seen in the livers from two of these animals (Fig. 4f).

**Fig. 5:**
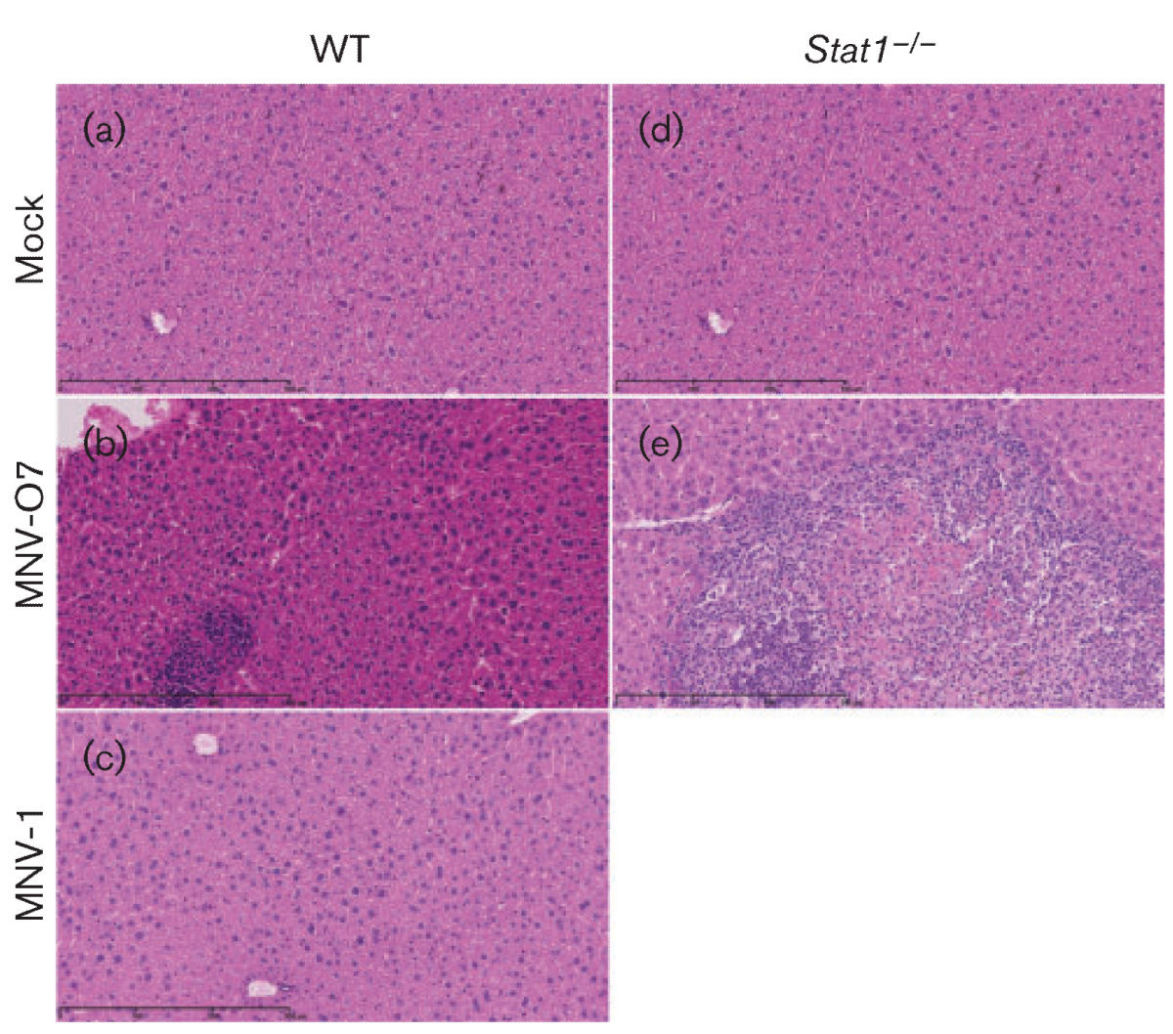
Histopathology in livers during chronic MNV infection. Haematoxylin & eosin-stained liver sections from mock (a, d) or infected mice (10^8^ RNA copies by oral gavage) at day 54 p.i. [MNV-O7 (b, e) and MNV-1 (c)]; infected WT (a–c) and *Stat1*^−/−^ (d, e) mice are shown. Original magnification, ×20 bar, 300 μm.

WT mice were kept until day 54 p.i. and no liver (Fig. 5a, mock; c, MNV-1) or splenic pathology (Fig. S8) was seen in mock- or MNV-1-infected mice.

After chronic infection (days 30–54 p.i.), no histopathological lesions were observed in the spleens of mock-infected WT or *Stat1*^−/−^ mice, nor in MNV-1- or MNV-O7-infected WT mice (Fig. S8). Interestingly, at this late time point in the spleens from *Stat1*^−/−^ mice infected with MNV-O7, there was red pulp hyperplasia and mild lymphoid hypoplasia and moderate to severe multifocal coalescing areas of inflammation with coagulative necrosis and fibrosis (Fig. S8). As expected, there was no histopathological lesions in the livers from mock-infected WT (Fig. 5a) and *Stat1*^−/−^ (Fig. 5d) mice. There were also no histopathological lesions in the livers from MNV-1-infected WT mice (Fig. 5c) at day 54 p.i.. In contrast, there were focal areas of mild perivascular inflammation consisting mainly of lymphocytes in the livers from MNV-O7-infected WT mice (Fig. 5b), whilst in *Stat1*^−/−^ mice infected with MNV-O7 there were large multifocal to coalescing areas of inflammation (mostly neutrophils), necrosis and fibrosis in the livers (Fig. 5e) – evidence of persistent ongoing inflammation caused by MNV-O7.

**Fig. 6:**
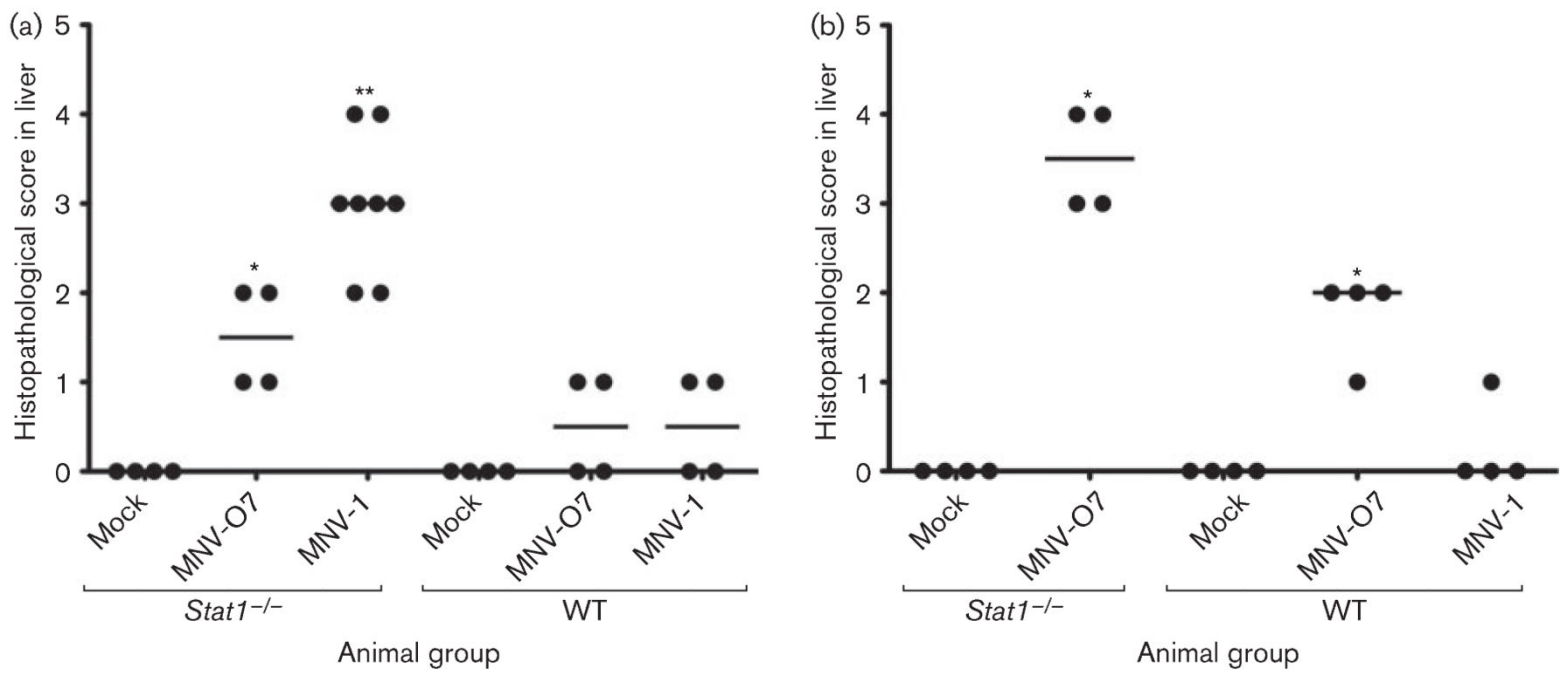
Histopathological grading of liver pathology from MNV-infected mice. Liver histopathology was scored (see Table S1 for scoring system) after (a) acute (day 7 p.i.) and (b) chronic (day 54 p.i.) infection (10^8^ RNA copies by oral gavage). Individual mouse scores and group medians are shown. Statistical analysis used a Mann–Whitney test; *P<0.01 and **P<0.001 comparing an infected group to the mock-infected group.

Histological lesions were scored in the liver (Fig. 6) and spleen (data not shown) at days 5 and 54 p.i. The lesion score in MNV-1-infected *Stat1*^−/−^ mice was increased when compared with mock-infected *Stat1*^−/−^ mice in the liver (*P*<0.01, Fig. 6a) and spleen (*P*<0.01) during acute infection. There was also an increase in lesion score in the livers of *Stat1*^−/−^ mice infected with MNV-O7 (*P*<0.05, Fig. 6a). Very few lesions were seen in WT mice with either virus at acute time points (Fig. 6a). During chronic infection, there was an increase in the lesion scores in MNV-O7-infected *Stat1*^−/−^ mice in the liver (*P*<0.05, Fig. 6b) and spleen (*P*<0.05) when compared with mock-inoculated *Stat1*^−/−^ mice. There was also a smaller but significant increase in liver lesions in MNV-O7-infected WT mice (*P*<0.05, Fig. 6b), whilst MNV-1-infected WT mice showed no increase in lesion score compared with mock-infected animals at late time points.

In a study of naturally infected immunodeficient mice, MNV infection caused hepatitis, focal interstitial pneumonia, peritonitis and pleuritis (Ward *et al.*, 2006). However, that study could not determine if the lesions were present after chronic infection because only naturally infected mice were investigated and so the time after infection was not known. In another study, MNV infection of immunocompetent Swiss Webster sentinel mice did not cause gross pathology; however, 80 % of the livers from 67 animals had small inflammatory foci, although this inflammation was not severe enough to be called hepatitis (Perdue *et al.*, 2007). No lesions were observed in the small intestine or spleen of animals in this study, but again the time of infection was unknown. Perdue *et al.* (2007) also showed that viral capsid antigen was present in liver lesions and Kupffer cells, and also in the mesenteric lymph nodes of these mice, suggesting active infection.

In summary, a subclinical variant of MNV was derived from *Stat1*^−/−^ mice and characterized genetically. Virus was generated directly from infected faeces by reverse genetics, and the molecularly cloned variant confirmed subclinical but ongoing pathology in *Stat1*^−/−^ mice and persistent infection in WT mice.

## METHODS

### Genotyping

Mouse genomic DNA was extracted with the DNeasy Blood and Tissue Kit (Qiagen) following the manufacturer’s instructions. Two separate PCRs were used to confirm the genetic status of the mice used. The first PCR (Agrawal *et al.*, 2007) distinguished WT, heterozygous and *Stat1*^−/−^ mice using three different primers: primer SF2992 (forward primer ctaccagagtatctgcctagac), primer SR3318 (reverse primer cctctcaaccttcctgacacc) and primer NeoR3392 (reverse primer cgccgctcccgattcgcagcgcatcgc). The second PCR used two primers (SF2893: forward primer gagggaatgtgtgatgggtcagg; SR4006: reverse primer attctccagagaaaagcggctgt) and distinguished mice bearing the *Stat1* gene (WT and heterozygous mice) from knockout mice (*Stat1*^−/−^ mice) (Mohan *et al.*, 2000).

### Derivation of a full-length molecular clone from faecal RNA

The full-length sequence of MNV from a WT mouse (MNV-O1) was obtained using primer walking, and used to design primers at the 5′ and 3′ ends of the genome (forward primer atagtttaggactagttaatacgactcactatagtgaaatgaggatggcaacgccatcttctgcgccc; reverse primer tcgcgaactagttttttttttttttttttttttttttaaaatgcatctaaatactactaaaagaaaagcagt). Using these primers, a full-length cDNA was derived directly from RNA extracted from a faecal sample obtained from an infected *Stat1*^−/−^ mouse (MNV-O7). RNA was reverse transcribed using random hexamers and SuperScript III (Invitrogen), and the PCR used the Roche Applied Science Long Ranger PCR Kit.

### Sequence analysis

Viral nucleotide sequences obtained (GenBank accession nos. KF113527 and KF113526 for MNV-O1 and MNV-O7, respectively) and their predicted amino acid sequences were aligned using Clustal W with full-length MNV genomic or protein sequences drawn from GenBank. A PhyML (Guindon *et al.*, 2010) phylogenetic tree was constructed using SeaView software with 100 bootstrap repetitions (http://pbil.univ-lyon1.fr/software/seaview.html; Gouy *et al.*, 2010). The tree was then edited using FigTree software (http://tree.bio.ed.ac.uk/software/figtree; Andrew Rambaut, Institute of Evolutionary Biology, University of Edinburgh, UK).

### Reverse genetics

The full-length MNV-O7 cDNA clone was inserted into pT_7_3′Rz to generate pT7-MNV-O7-Rz carrying the O7 sequence under the control of a T7 RNA polymerase promoter. Using site-directed mutagenesis, the *Spe*I restriction site sequence on the 3′ primer was altered to a *Nhe*I restriction site sequence and the plasmid used to recover infectious virus as described previously (Chaudhry *et al.*, 2007). Molecular clones of MNV-1.CW1, MNV-3, PIb (infectious MNV-1 produced by reverse genetics) and FS (MNV-1 construct with a frameshift) were used as controls (Arias *et al.*, 2012; Chaudhry *et al.*, 2007).

### Virus culture and quantification

MNV-1.CW3 was kindly provided by Professor H. Virgin (Department of Pathology and Immunology, Washington State University, USA) and is not persistent in WT mice, but is pathogenic in *Stat1*^−/−^ mice (Mumphrey *et al.*, 2007). MNV-O7 was cultured from homogenized faecal samples from a *Stat1*^−/−^ mouse grown on RAW264.7 cells for 4 days, and the clarified supernatant taken after freeze–thawing and filtering through a 0.22 μm filter, and stored at −80 °C. MNV-O7 was amplified briefly by one passage on RAW264.7 cells. MNV was used to infect RAW264.7 cells at a multiplicity of 0.1 TCID_50_ cell^−1^. Cells were incubated at 37 °C until CPEs approached 100 % or after 3–4 days if CPEs were not obvious. The virus was harvested and stored as above. Virus titre was determined by measuring TCID_50_ in RAW264.7 cells (if the virus induced CPEs) or by qRT-PCR.

To quantify the amount of virus in mouse faeces, tissues or cell culture, a real-time RT-qPCR was performed using a dual-labelled probe (forward primer, FQ2: cgctgcgccatcactcatc; reverse primer, RQ2: gctttggaacaatggatgctgag; probe2: FAM-ccgcaggaacgctcagcagtctt-BHQ1). RNA was extracted using the Roche Applied Science High Pure Viral RNA Kit and reverse transcribed as above. One-tenth of the cDNA reaction was used in the real-time PCR with Quantitect Probe PCR Master Mix using the manufacturer’s suggested primer and probe concentrations, and the PCR performed in a Rotor-Gene 6000 (Qiagen): *Taq* activation (15 min, 95 °C); PCR 40 cycles: 10 s, 95 °C, 30 s, 60 °C; in triplicate. A standard curve was made using MNV-O7 cDNA cut from pT7-MNV-O7-Rz using *Spe*I and *Nhe*I. The gel-purified DNA was diluted in 10 μg carrier RNA (yeast tRNA; Roche Applied Science) ml^−1^ in water to obtain 5×10^11^ to 5 molecules μl^−1^. The assay was validated using these DNA standards and RNA dilutions from infected tissue culture. Data were analysed using the Rotor-Gene 6000 series software 1.7 (Qiagen). The efficiency of the RT-qPCR for both RNA and DNA standard curves was unity. The range of the assay was 50 to 5×10^10^ copies per reaction.

### ELISA for antibodies

Diagnostic MNV ELISA microtitre plates (Charles River Laboratories) were used as per the manufacturer’s instructions with 50 μl serum diluted 1: 60 (derived from a test serum titration). Positive and negative (CL550 and CL500, respectively; Charles River Laboratories) serum controls were used with each ELISA plate.

### Animals and *in vivo* infections

*Stat1* knockout (129S6/SvEv-*Stat1*^*tm1Rds*^, here *Stat1*^−/−^), WT (129S6/SvEv) and heterozygous mice were obtained originally from Taconic Farms. A small mix-breed group of animals from the John Radcliffe Hospital, University of Oxford was shipped to Cambridge for further study. The mice had no history of clinical disease and were free of known murine pathogens as listed on the Federation of European Laboratory Animal Science Associations guidelines (Nicklas *et al.*, 2002) with the exception of MNV. The mice were ear biopsied for identification purposes and the biopsies used to extract DNA for genotyping (see above). From the mixed group, mice were rederived, genotyped and bred to homozygosity for the *Stat1* gene deletion. Similarly, *Stat1*-positive animals were bred to homozygosity to use as WT immunocompetent animals. For experimental infections, mice were used at 8–12 weeks of age and were negative for anti-MNV antibody by ELISA. The mice were housed in individually ventilated cages, and fed sterilized water and diet. Housing, care and procedures were carried out in compliance with the University ethical review process and the Animals (Scientific Procedures) Act 1986. Animals were inoculated with virus by oral gavage. Faecal samples were collected from individual mice.

### Gross pathology

The following tissues were examined for gross lesions: intestine, mesenteric lymph node, spleen, liver and lung. The liver, spleen and lung were measured by ruler and digital images taken.

### Histopathology

Small pieces of the small intestine (duodenum, jejunum and ileum), caecum, colon, mesenteric lymph node, spleen, liver and lung were placed in 10 % buffered formal saline and embedded in paraffin. Haematoxylin and eosin-stained 3 μm thick sections were viewed with a Carl Zeiss AxioVision microscope, the images captured using an AxioCam camera and processed using the AxioVision digital image processing software and/or the sections captured with a digital slide scanner Hamamatsu NanoZoomer 2.0 RS and the images viewed with NDP.view2.

The tissues were graded for the degree of inflammation, fibrosis and necrosis using a modification of published grading systems (Hübscher, 1998; Theise, 2007; Wirtz *et al.*, 2007) (Table S1, 10 fields per section were scored). The following were used: in the liver, hepatitis characterized by focal or multifocal infiltration of inflammatory cells mainly composed of mononuclear cells, macrophage-like cells and neutrophils with or without associated necrosis and/or fibrosis; in the lungs, pneumonia with focal or multifocal infiltration of mononuclear cells, macrophage-like cells and neutrophils in alveoli and alveolar walls with or without associated necrosis and/or fibrosis; in the mesenteric lymph node, activation, necrosis and/or fibrosis; in the blood vessel, vasculitis with adhesions of mononuclear cells and leukocytes to hepatic and pulmonary veins; in the spleen, activation, necrosis and/or fibrosis.

## Supplementary Material

Supplementary Table and Figures

## Figures and Tables

**Table 1:** Diagnostic viral RT-PCRs of tissue and faecal samples from MNV-infected mice

Day p.i.	Tissue	Mouse type	Inoculum		
			Medium	MNV-O7	MNV-1
5	Faeces	*Stat1* ^−/−^	0/8	8/8	8/8
		WT	0/8	8/8	8/8
54	Faeces	*Stat1* ^−/−^	nd	0/5	na
		WT	nd	4/4	0/4
54	Liver	*Stat1* ^−/−^	nd	2/4	na
		WT	nd	4/4	0/4
54	Spleen	*Stat1* ^−/−^	nd	3/4	na
		WT	nd	4/4	0/4

nd, Not done; na, not applicable.

## References

[R1] Agrawal S, Febbraio M, Podrez E, Cathcart MK, Stark GR, Chisolm GM (2007). Signal transducer and activator of transcription 1 is required for optimal foam cell formation and atherosclerotic lesion development. Circulation.

[R2] Arias A, Bailey D, Chaudhry Y, Goodfellow I (2012). Development of a reverse-genetics system for murine norovirus 3: long-term persistence occurs in the caecum and colon. J Gen Virol.

[R3] Bailey D, Thackray LB, Goodfellow IG (2008). A single amino acid substitution in the murine norovirus capsid protein is sufficient for attenuation in vivo. J Virol.

[R4] Capizzi T, Makari-Judson G, Steingart R, Mertens WC (2011). Chronic diarrhea associated with persistent norovirus excretion in patients with chronic lymphocytic leukemia: report of two cases. BMC Infect Dis.

[R5] Chachu KA, LoBue AD, Strong DW, Baric RS, Virgin HW (2008a). Immune mechanisms responsible for vaccination against and clearance of mucosal and lymphatic norovirus infection. PLoS Pathog.

[R6] Chachu KA, Strong DW, LoBue AD, Wobus CE, Baric RS, Virgin HW (2008b). Antibody is critical for the clearance of murine norovirus infection. J Virol.

[R7] Chaudhry Y, Skinner MA, Goodfellow IG (2007). Recovery of genetically defined murine norovirus in tissue culture by using a fowlpox virus expressing T7 RNA polymerase. J Gen Virol.

[R8] Coyne KP, Dawson S, Radford AD, Cripps PJ, Porter CJ, McCracken CM, Gaskell RM (2006). Long-term analysis of feline calicivirus prevalence and viral shedding patterns in naturally infected colonies of domestic cats. Vet Microbiol.

[R9] Coyne KP, Gaskell RM, Dawson S, Porter CJ, Radford AD (2007). Evolutionary mechanisms of persistence and diversification of a calicivirus within endemically infected natural host populations. J Virol.

[R10] Forrester NL, Boag B, Moss SR, Turner SL, Trout RC, White PJ, Hudson PJ, Gould EA (2003). Long-term survival of New Zealand rabbit haemorrhagic disease virus RNA in wild rabbits, revealed by RT-PCR and phylogenetic analysis. J Gen Virol.

[R11] Forrester NL, Moss SR, Turner SL, Schirrmeier H, Gould EA (2008). Recombination in rabbit haemorrhagic disease virus: possible impact on evolution and epidemiology. Virology.

[R12] Gil MP, Bohn E, O’Guin AK, Ramana CV, Levine B, Stark GR, Virgin HW, Schreiber RD (2001). Biologic consequences of Stat1-independent IFN signaling. Proc Natl Acad Sci U S A.

[R13] Godinez I, Raffatellu M, Chu H, Paixão TA, Haneda T, Santos RL, Bevins CL, Tsolis RM, Baümler AJ (2009). Interleukin-23 orchestrates mucosal responses to Salmonella enterica serotype Typhimurium in the intestine. Infect Immun.

[R14] Gouy M, Guindon S, Gascuel O (2010). SeaView version 4: a multiplatform graphical user interface for sequence alignment and phylogenetic tree building. Mol Biol Evol.

[R15] Guindon S, Dufayard JF, Lefort V, Anisimova M, Hordijk W, Gascuel O (2010). New algorithms and methods to estimate maximum-likelihood phylogenies: assessing the performance of PhyML 3.0. Syst Biol.

[R16] Hsu CC, Riley LK, Wills HM, Livingston RS (2006). Persistent infection with and serologic cross-reactivity of three novel murine noroviruses. Comp Med.

[R17] Hsu CC, Riley LK, Livingston RS (2007). Molecular characterization of three novel murine noroviruses. Virus Genes.

[R18] Hübscher SG (1998). Histological grading and staging in chronic hepatitis: clinical applications and problems. J Hepatol.

[R19] Johnson RP (1992). Antigenic change in feline calicivirus during persistent infection. Can J Vet Res.

[R20] Kahan SM, Liu G, Reinhard MK, Hsu CC, Livingston RS, Karst SM (2011). Comparative murine norovirus studies reveal a lack of correlation between intestinal virus titers and enteric pathology. Virology.

[R21] Karst SM, Wobus CE, Lay M, Davidson J, Virgin HW (2003). STAT1-dependent innate immunity to a Norwalk-like virus. Science.

[R22] Katpally U, Wobus CE, Dryden K, Virgin HW, Smith TJ (2008). Structure of antibody-neutralized murine norovirus and unexpected differences from viruslike particles. J Virol.

[R23] Lochridge VP, Hardy ME (2007). A single-amino-acid substitution in the P2 domain of VP1 of murine norovirus is sufficient for escape from antibody neutralization. J Virol.

[R24] Malmgaard L, Salazar-Mather TP, Lewis CA, Biron CA (2002). Promotion of alpha/beta interferon induction during in vivo viral infection through alpha/beta interferon receptor/STAT1 system-dependent and -independent pathways. J Virol.

[R25] Manuel CA, Hsu CC, Riley LK, Livingston RS (2008). Soiled-bedding sentinel detection of murine norovirus 4. J Am Assoc Lab Anim Sci.

[R26] McFadden N, Arias A, Dry I, Bailey D, Witteveldt J, Evans DJ, Goodfellow I, Simmonds P (2013). Influence of genome-scale RNA structure disruption on the replication of murine norovirus – similar replication kinetics in cell culture but attenuation of viral fitness *in vivo*. Nucleic Acids Res.

[R27] Mohan RR, Mohan RR, Kim WJ, Stark GR, Wilson SE (2000). Defective keratocyte apoptosis in response to epithelial injury in Stat 1 null mice. Exp Eye Res.

[R28] Mumphrey SM, Changotra H, Moore TN, Heimann-Nichols ER, Wobus CE, Reilly MJ, Moghadamfalahi M, Shukla D, Karst SM (2007). Murine norovirus 1 infection is associated with histopathological changes in immunocompetent hosts, but clinical disease is prevented by STAT1-dependent interferon responses. J Virol.

[R29] Nice TJ, Strong DW, McCune BT, Pohl CS, Virgin HW (2013). A single-amino-acid change in murine norovirus NS1/2 is sufficient for colonic tropism and persistence. J Virol.

[R30] Nicklas W, Baneux P, Boot R, Decelle T, Deeny AA, Fumanelli M, Illgen-Wilcke B, FELASA (Federation of European Laboratory Animal Science Associations Working Group on Health Monitoring of Rodent and Rabbit Colonies) (2002). Recommendations for the health monitoring of rodent and rabbit colonies in breeding and experimental units. Lab Anim.

[R31] Oldstone MB (2006). Viral persistence: parameters, mechanisms and future predictions. Virology.

[R32] Radford AD, Turner PC, Bennett M, McArdle F, Dawson S, Glenn MA, Williams RA, Gaskell RM (1998). Quasispecies evolution of a hypervariable region of the feline calicivirus capsid gene in cell culture and in persistently infected cats. J Gen Virol.

[R33] Ramana CV, Gil MP, Schreiber RD, Stark GR (2002). Stat1-dependent and -independent pathways in IFN-gamma-dependent signaling. Trends Immunol.

[R34] Shresta S, Sharar KL, Prigozhin DM, Snider HM, Beatty PR, Harris E (2005). Critical roles for both STAT1-dependent and STAT1-independent pathways in the control of primary dengue virus infection in mice. J Immunol.

[R35] Siebenga JJ, Beersma MF, Vennema H, van Biezen P, Hartwig NJ, Koopmans M (2008). High prevalence of prolonged norovirus shedding and illness among hospitalized patients: a model for in vivo molecular evolution. J Infect Dis.

[R36] Taube S, Rubin JR, Katpally U, Smith TJ, Kendall A, Stuckey JA, Wobus CE (2010). High-resolution x-ray structure and functional analysis of the murine norovirus 1 capsid protein protruding domain. J Virol.

[R37] Thackray LB, Wobus CE, Chachu KA, Liu B, Alegre ER, Henderson KS, Kelley ST, Virgin HW (2007). Murine noroviruses comprising a single genogroup exhibit biological diversity despite limited sequence divergence. J Virol.

[R38] Theise ND (2007). Liver biopsy assessment in chronic viral hepatitis: a personal, practical approach. Mod Pathol.

[R39] Tomov VT, Osborne LC, Dolfi DV, Sonnenberg GF, Monticelli LA, Mansfield K, Virgin HW, Artis D, Wherry EJ (2013). Persistent enteric murine norovirus infection is associated with functionally suboptimal virus-specific CD8 T cell responses. J Virol.

[R40] Ward JM, Wobus CE, Thackray LB, Erexson CR, Faucette LJ, Belliot G, Barron EL, Sosnovtsev SV, Green KY (2006). Pathology of immunodeficient mice with naturally occurring murine norovirus infection. Toxicol Pathol.

[R41] Wirtz S, Neufert C, Weigmann B, Neurath MF (2007). Chemically induced mouse models of intestinal inflammation. Nat Protoc.

[R42] Wobus CE, Karst SM, Thackray LB, Chang KO, Sosnovtsev SV, Belliot G, Krug A, Mackenzie JM, Green KY, Virgin HW (2004). Replication of Norovirus in cell culture reveals a tropism for dendritic cells and macrophages. PLoS Biol.

