# Supplementary material for: Pathology caused by persistent murine norovirus infection
Source: J Gen Virol. Author manuscript; Available in PMC 2015 Jan 29. (PMC4310207; doi:10.1099/vir.0.059188-0)
Supplement: Supplementary Table and Figures [file NIHMS61893-supplement-WT089214.pdf]

Table S1. Scoring system for histological changes

Score	Histological changes*
0	No evidence of inflammation, normal architecture
1	Mild inflammation with 1-2 foci of inflammatory cells per 10 fields at 10X objective
2	Moderate inflammation with 5 to 10 foci of inflammatory cells per 10 fields at 10X objective
3	Severe inflammation with more than 10 foci of inflammatory cells per 10 fields at 10X objective
4	Moderate inflammation with focal areas of necrosis
5	Moderate inflammation with diffuse necrosis and fibrosis
6	Severe inflammation with focal areas of necrosis
7	Severe inflammation with multifocal and diffuse necrosis and fibrosis

* The following background changes in the tissues were noted but were not included in the final grading: iron or haemosiderin deposition in the spleen and/or liver, presence of multinucleated hepatocytes, glycogen deposition in hepatocytes and extramedullary erythropoiesis in the spleen and liver.

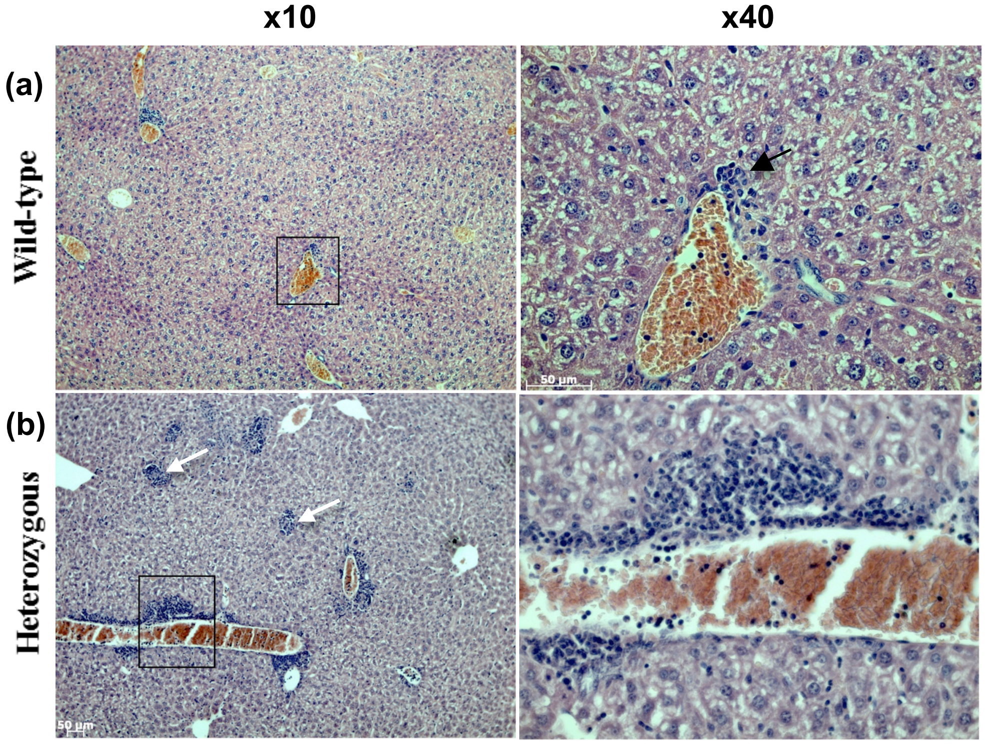

Figure S1. Liver histopathology in naturally infected mice
Representative pathology in liver sections from mice naturally infected with MNV-O7. Haematoxylin and eosin staining of formaldehyde-fixed paraffin embedded tissues. (a) WT and (b) heterozygous mice. Magnifications as indicated. (a) x40 view shows vasculitis (arrow) and (b) x10 view shows multifocal, diffuse areas of inflammation (white arrows) and x40 view shows an area of necrosis and fibrosis around a blood vessel.

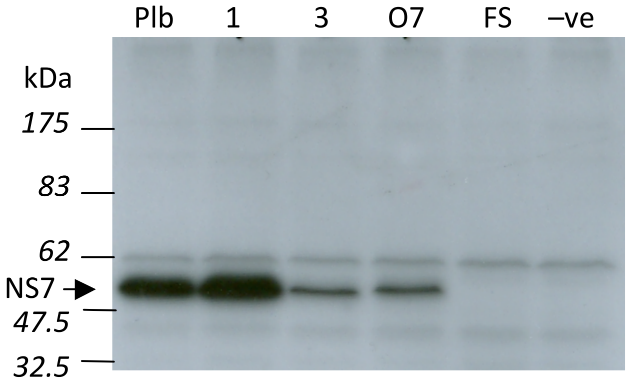

Figure S2. Anti-NS7 western blot of molecular clone transfected cell supernatants BSR-T7 cells were transfected with murine norovirus molecular clones MNV-1 (1), MNV-3 (3), MNV-O7 (O7), a frameshifted MNV-1 (FS) or were untransfected (-ve) or infected with MNV-1 derived from a molecular clone (PIb). Supernatants were taken 7 days post transfection, separated on a SDS-PAGE gel and blotted onto Immobilon transfer membranes. The blots were probed with rabbit anti-NS7 serum. NS7 is seen as an approximately 50 kDa band.

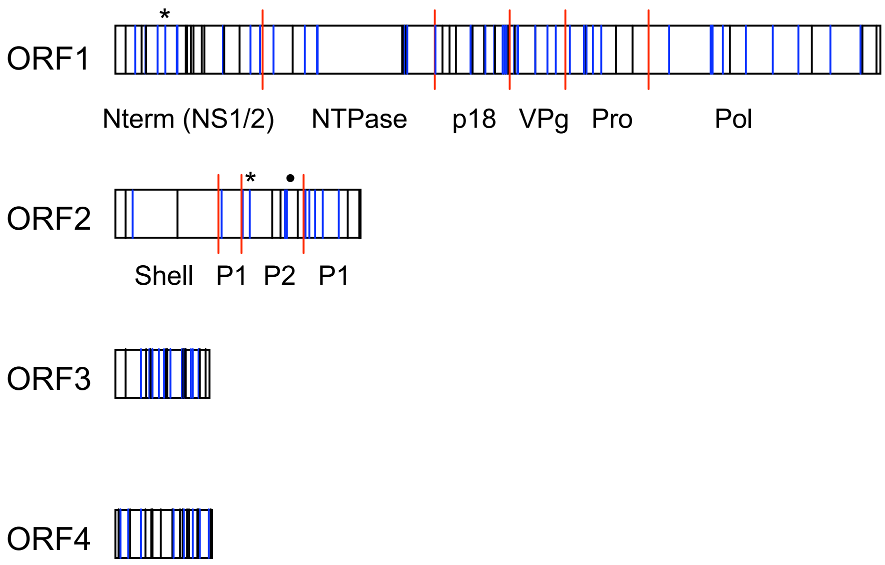

Figure S3. Open reading frames of MNV.
The amino acid differences between MNV-O7 and -1 in the 4 open reading frames of MNV are shown. Black lines represent non-conservative changes, blue conservative changes. In ORF1, red lines show the predicted cleavage sites of the polyprotein. The cleavage site between NTPase and p18 is QS in MNV-O7 and QN in MNV-1. In ORF2, red lines represent the domain boundaries of VP1. The star represents amino acid changes associated with persistence (ORF1) and virulence (ORF2). The dot represents the site of the Mab A6.2 epitope in VP1.

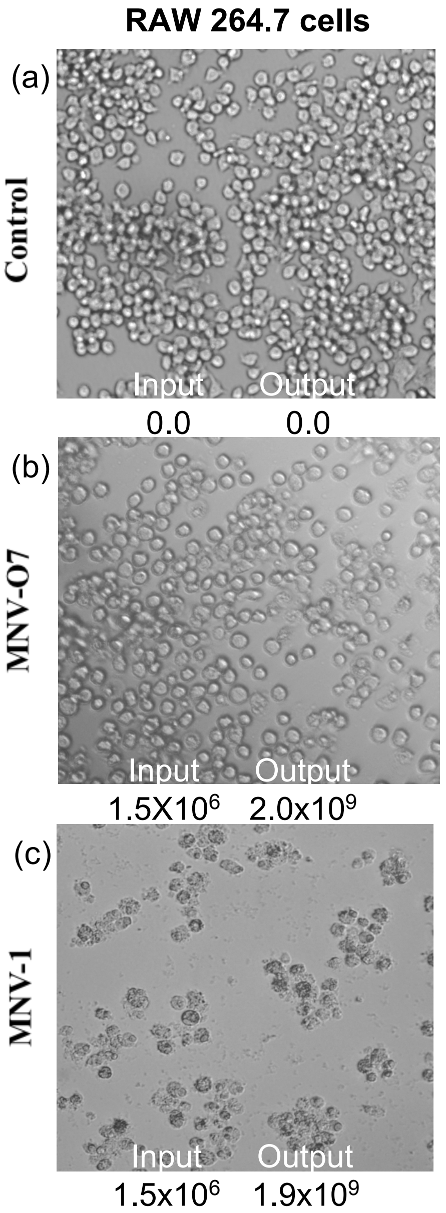

Figure S4. MNV-O7 was non-cytopathic in RAW264.7 cells

RAW264.7 cells were mock infected or infected with 1.5 x 10^6 RNA copies of MNV-O7 or MNV-1 and the cells incubated for 4 days. Virus was titred by quantitative RT-PCR and the total number of genome copies put into the culture and then recovered after 4 days are indicated. Phase contrast images are shown at x20 magnification.

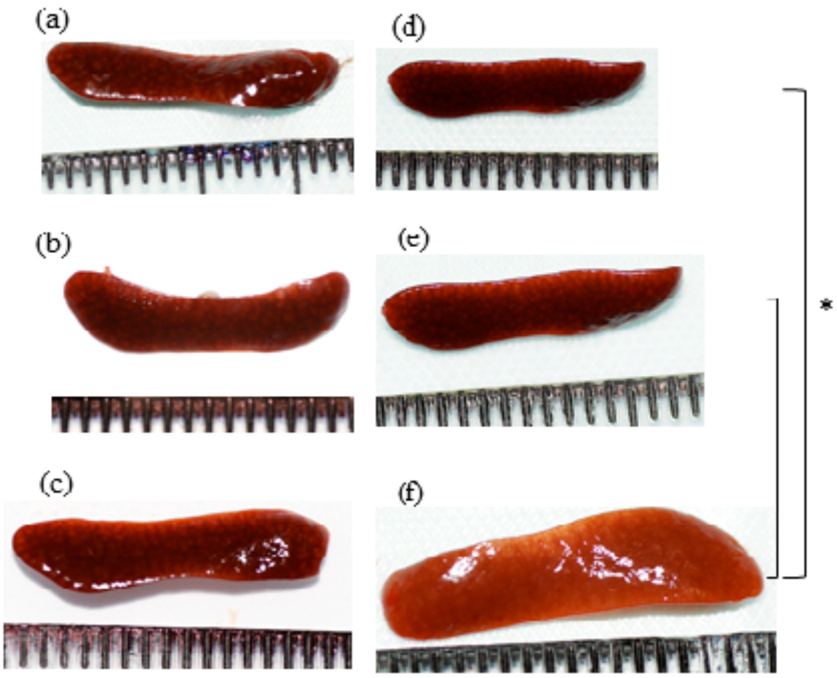

Figure S5. Gross pathology in spleens from acutely infected mice
Mice were infected with 10^8 RNA copies of MNV by oral gavage and spleens taken at day 5 p.i.. The spleens from wild-type mice (a to c) and *Stat1*$^{-/-}$ mice (d to f) are shown next to a mm scale. Mock (a and d**)**, MNV-O7 (b and e) and MNV-1 (c and f) infected animals. P values were determined using one-way ANOVA on the median spleen length. * represents $p < 0.05$.

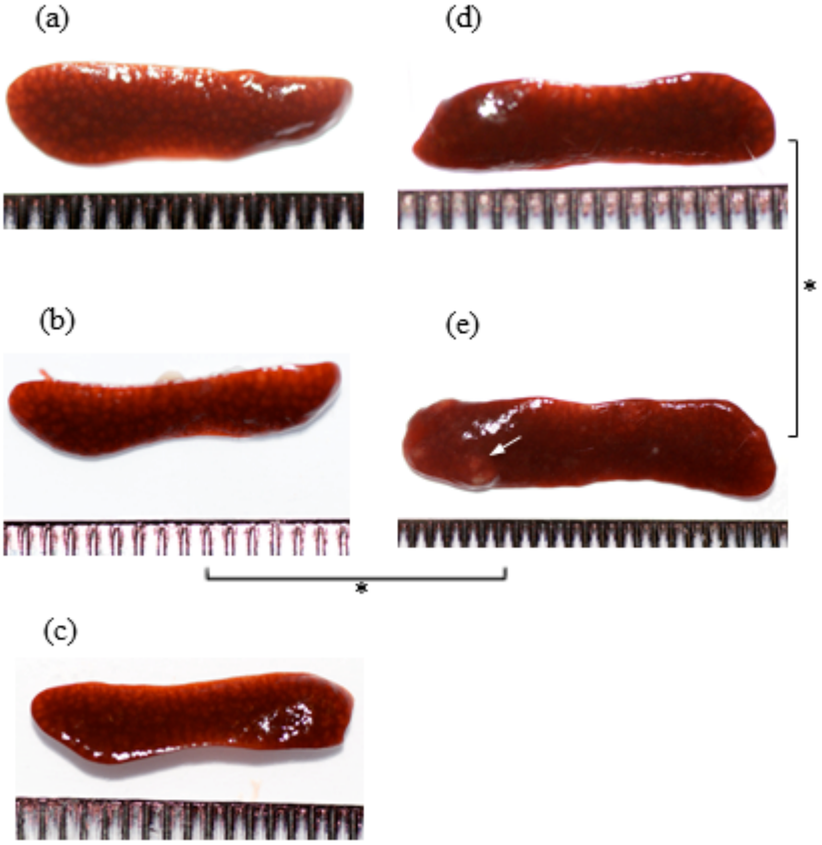

Figure S6. Gross pathology in spleens from chronically infected mice
Mice were infected with 10^8 RNA copies of MNV by oral gavage and spleens taken at day 54 p.i.. The spleens from wild-type mice (a to c) and *Stat1*$^{-/-}$ mice (d, e) are shown next to a mm scale. Mock (a and d**),** MNV-O7 (b and e) and MNV-1 (c) infected animals. P values were determined using one-way ANOVA on the median spleen length. * represents $p < 0.05$.

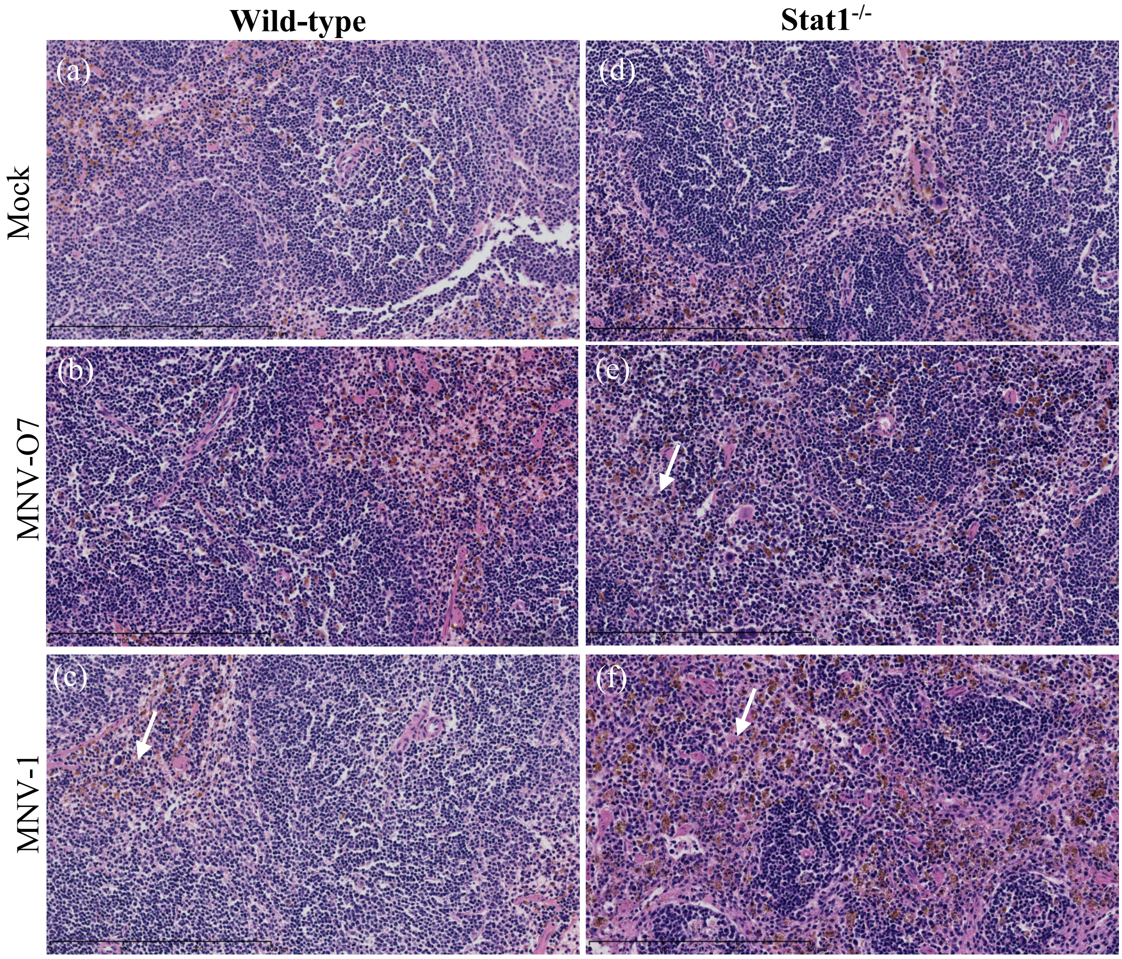

Figure S7. Histopathology in spleens during acute MNV infection
Haematoxylin and eosin stained spleen sections from mock (a and d) or infected mice (10^8 RNA copies by oral gavage) at day 5 p.i.; MNV-O7 (b and e) and MNV-1 (c and f): infected WT (a to c) and *Stat1*$^{-/-}$ (d to f) mice are shown. Arrows show red pulp expansion (c, e) and coagulative necrosis (f). All at x20 magnification, scale bar shows 300 um.

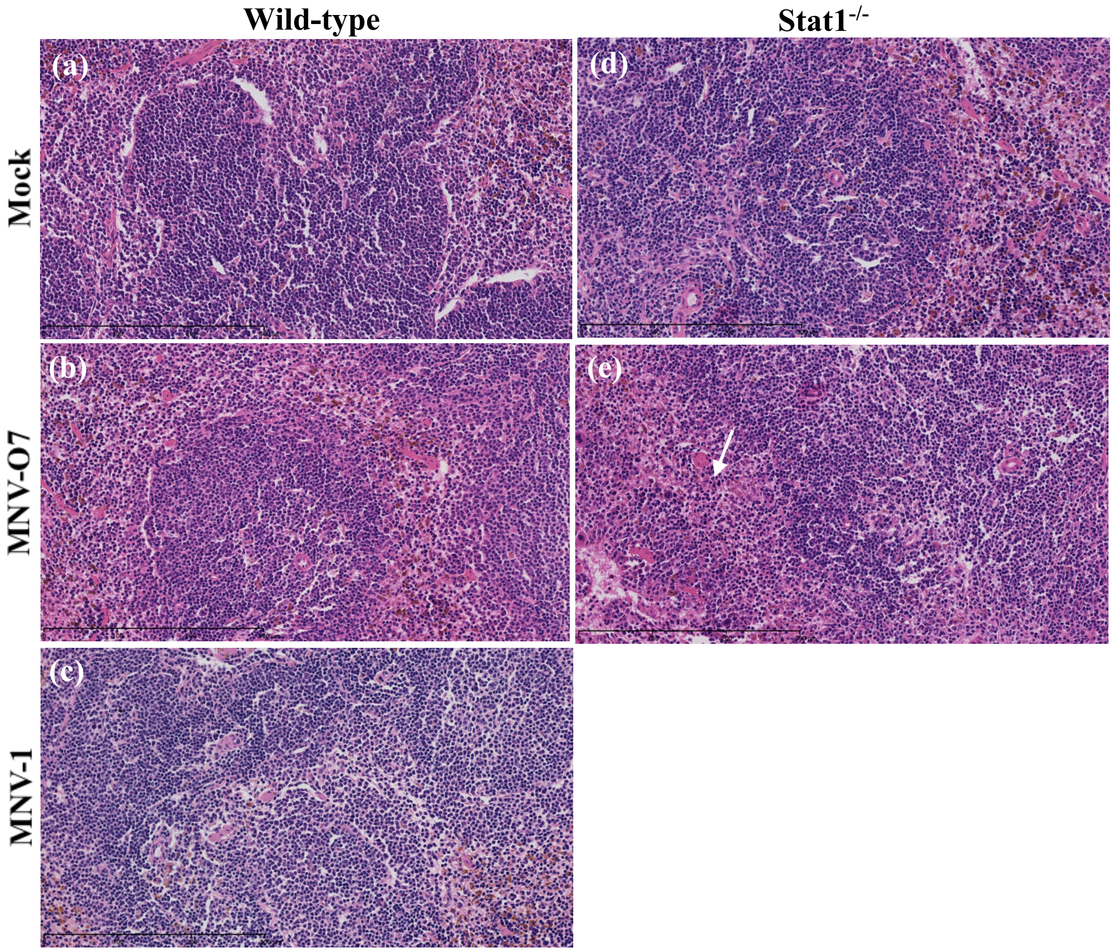

Figure S8. Histopathology in spleens during chronic MNV infection
Haematoxylin and eosin stained spleen sections were taken from mock (a and d) or infected mice (10^8 RNA copies by oral gavage) at day 54 p.i.; MNV-O7 (b and e) and MNV-1 (c): infected WT (a to c) and *Stat1*$^{-/-}$ (d and e) mice are shown. The arrow shows coagulative necrosis and fibrosis (e). All at x20 magnification, scale bar shows 300 um.
